# Hypocretin1/orexinA-immunoreactive axons form few synaptic contacts on rat ventral tegmental area neurons that project to the medial prefrontal cortex

**DOI:** 10.1186/1471-2202-15-105

**Published:** 2014-09-07

**Authors:** Esther Del Cid-Pellitero, Miguel Garzón

**Affiliations:** Departamento de Anatomía, Histología y Neurociencia, Facultad de Medicina, Universidad Autónoma de Madrid, Arzobispo Morcillo 4, 28029 Madrid, Spain; Instituto de Investigación Hospital Universitario La Paz (IDIPAZ), Paseo de la Castellana 261, 28046 Madrid, Spain

**Keywords:** Cortical activation, Sleep, Ultrastructure, Wakefulness, Dopamine, GABA

## Abstract

**Background:**

Hypocretins/orexins (Hcrt/Ox) are hypothalamic neuropeptides involved in sleep-wakefulness regulation. Deficiency in Hcrt/Ox neurotransmission results in the sleep disorder narcolepsy, which is characterized by an inability to maintain wakefulness. The Hcrt/Ox neurons are maximally active during wakefulness and project widely to the ventral tegmental area (VTA). A dopamine-containing nucleus projecting extensively to the cerebral cortex, the VTA enhances wakefulness. In the present study, we used retrograde tracing from the medial prefrontal cortex (mPFC) to examine whether Hcrt1/OxA neurons target VTA neurons that could sustain behavioral wakefulness through their projections to mPFC.

**Results:**

The retrograde tracer Fluorogold (FG) was injected into mPFC and, after an optimal survival period, sections through the VTA were processed for dual immunolabeling of anti-FG and either anti-Hcrt1/OxA or anti-TH antisera. Most VTA neurons projecting to the mPFC were located in the parabrachial nucleus of the ipsilateral VTA and were non-dopaminergic. Only axonal profiles showed Hcrt1/OxA-immunoreactivity in VTA. Hcrt1/OxA reactivity was observed in axonal boutons and many unmyelinated axons. The Hcrt1/OxA immunoreactivity was found filling axons but it was also observed in parts of the cytoplasm and dense-core vesicles. Hcrt1/OxA-labeled boutons frequently apposed FG-immunolabeled dendrites. However, Hcrt1/OxA-labeled boutons rarely established synapses, which, when they were established, were mainly asymmetric (excitatory-type), with either FG-labeled or unlabeled dendrites.

**Conclusions:**

Our results provide ultrastructural evidence that Hcrt1/OxA neurons may exert a direct synaptic influence on mesocortical neurons that would facilitate arousal and wakefulness. The paucity of synapses, however, suggest that the activity of VTA neurons with cortical projections might also be modulated by Hcrt1/OxA non-synaptic actions. In addition, Hcrt1/OxA could modulate the postsynaptic excitatory responses of VTA neurons with cortical projections to a co-released excitatory transmitter from Hcrt1/OxA axons. Our observation of Hcrt1/OxA targeting of mesocortical neurons supports Hcrt1/OxA wakefulness enhancement in the VTA and could help explain the characteristic hypersomnia present in narcoleptic patients.

## Background

The ventral tegmental area (VTA) comprises several subdivisions including the paranigral (PN), parabrachial (PBP) or interfascicular (IF) nuclei [1]. Neurons synthesizing neurotransmitters, such as dopamine (DA), GABA or glutamate among others coexist in these VTA divisions [2, 3]. The mesocortical system, which is strongly involved in cognitive behavior, EEG activation and arousal, is formed by VTA projections to areas of the cerebral cortex located mainly in the medial surface of the frontal lobe [4]. VTA neurons projecting to the medial prefrontal cortex (mPFC) are distributed throughout both the PBP and PN [5]. Although much emphasis has been placed on the dopaminergic mesocortical system in previous studies, some reports indicate that GABA-containing VTA neurons also project to the mPFC [6]. Moreover, GABAergic VTA neurons are active during wakefulness [7]. VTA stimulation produces inhibition of cortical pyramidal neurons through activation of both GABA cortical interneurons and GABA mesocortical VTA neurons [8, 9]. However, systematic studies assessing the relative contributions of dopaminergic and non-dopaminergic neurons to the mesocortical pathway reaching mPFC have not been addressed so far.

Hypothalamic neurons containing the peptides Hypocretins/orexins (Hcrt/Ox) project widely to the VTA [10–12]. The Hcrt/Ox peptide family involved in sleep-wakefulness regulation comprises two peptides: Hcrt1/OxA and Hcrt2/OxB. Hcrt1/OxA binds to Hcrt/Ox-R1 and Hcrt/Ox-R2, both of which are present in VTA neurons [13]. Deficient actions of Hcrt/Ox due to either degeneration of Hcrt/Ox neurons or breakdown of Hcrt/Ox signaling pathways result in the chronic sleep disorder narcolepsy, which is characterized by inability to maintain prolonged periods of wakefulness [14–16]. Furthermore, Hcrt/Ox neurons discharge maximally in wakefulness, decrease their activity in slow wave sleep and cease firing during REM sleep [17, 18].

Intracerebroventricular Hcrt/Ox increase mPFC DA release and neuronal activity, and thereby arousal [19, 20]. Moreover, Hcrt1/OxA infusion in the VTA increases wakefulness and decreases sleep [21]. Hcrt1/OxA application *in vitro* enhances VTA neuron activity [22], suggesting that Hcrt1/OxA excites the mPFC partly though activation of mesocortical VTA neurons.

A 2007 study has reported the existence of only a few synapses between Hcrt1/OxA axons and VTA neurons; some of these neurons were identified as either dopaminergic or GABAergic [23]. Thus, Hcrt1/OxA could affect VTA function synaptically, but also perhaps through non-synaptic actions that have been reported typical of peptidergic transmission [24, 25]. Here we hypothesize that some of those neurons targeted by Hcrt1/OxA axons belong to the mesocortical pathway. Nevertheless, there are no ultrastructural studies about particular relationships between Hcrt1/OxA axons and VTA neurons projecting unambiguously to mPFC, and the definite targeting of mesocortical neurons by Hcrt1/OxA axons has not been assessed yet.

Here we determine: 1) the comparative contribution of VTA dopaminergic and non-dopaminergic neurons to mesocortical projections to mPFC; 2) the ultrastructural distribution of Hcrt1/OxA in the VTA; and 3) the cellular relationships of Hcrt1/OxA-containing axons with VTA neurons projecting to mPFC. The results obtained identify ultrastructural bases for hypocretinergic activation of VTA mesocortical neurons and improveour understanding of the anatomical linkages supporting the well-known wake-enhancing and cortical activation actions of Hcrt1/OxA at the VTA.

## Results

### Injection sites

**Figure 1:**
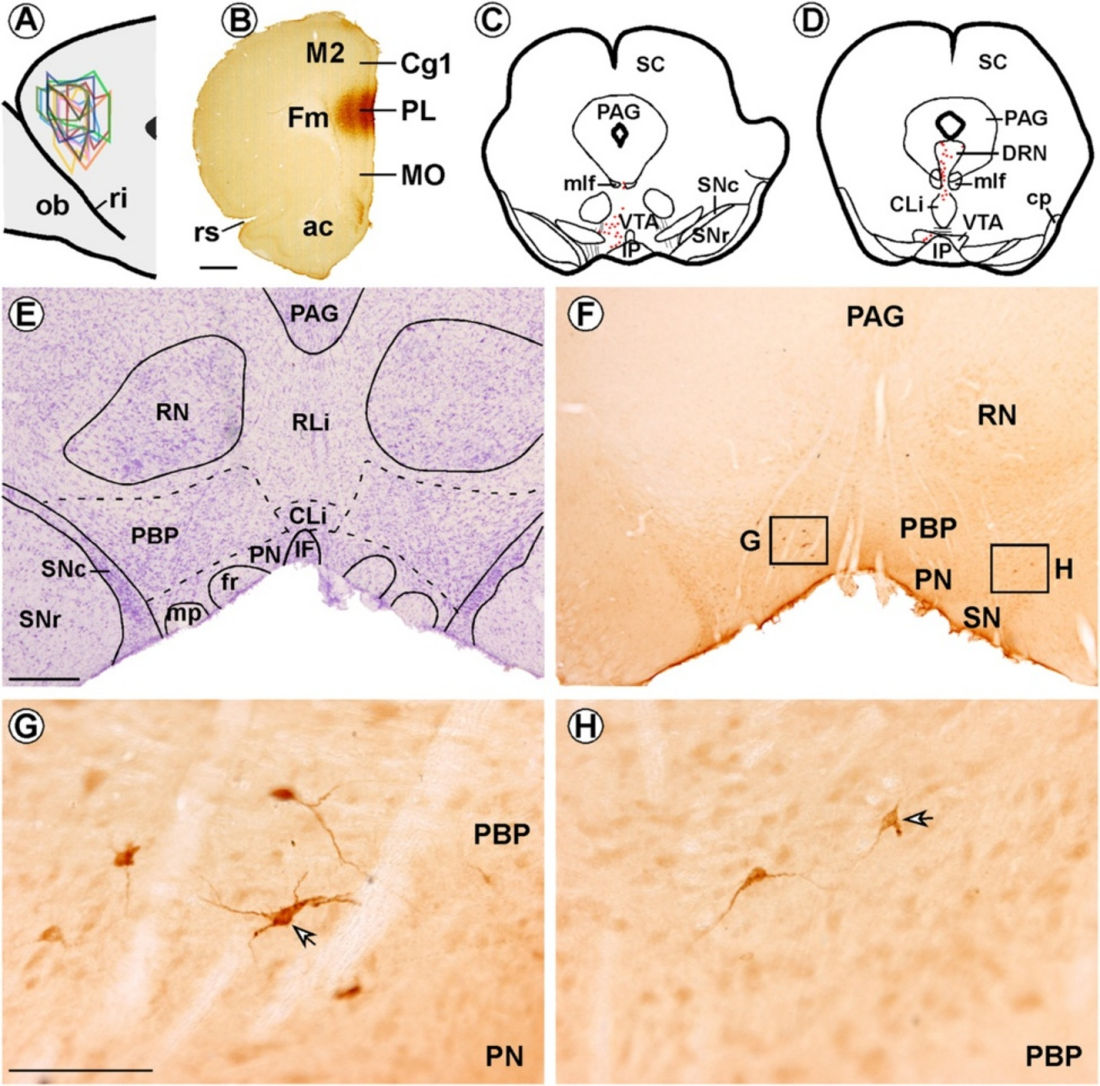
**Location of Fluorogold (FG) injections in the prelimbic region (PL) of the medial prefrontal cortex and FG-labeled neurons in the ventral tegmental area (VTA). (A)** Sagittal scheme shows all FG infusions in PL (n = 10; modified from Swanson, 1998). **(B)** Coronal section showing an FG deposit in PL using immunohistochemistry in one animal. **(C-D)** Representative coronal brainstem drawings showing FG-immunolabeled neurons (red dots) in the VTA of one of the PL group rats. **(E)** Nissl-stained section adjacent to the section shown in F, which delineates the subdivisions of VTA. **(F)** Panoramic photomicrograph showing FG-labeled neurons in the parabrachial subdivision (PBP) of VTA. **(G)** High magnification of box “G” from **F** showing FG-labeled neurons in the PBP of VTA ipsilateral to the FG injection site. The peroxidase immunoreaction product can be clearly observed in the cytoplasm of cell bodies and proximal dendritic branches (arrow). **(H)** High magnification of box “H” from **F**, showing much weaker FG-retrograde labeling (arrow) in the PBP of VTA contralateral to the FG injection. ac, anterior commissure; Cg1, cingular cortex, CLi, caudal linear raphe nucleus; DRN, dorsal raphe nucleus; Fm, forceps minor of the corpus callosum; fr, fasciculus retroflexus; IF, interfascicular subdivision of VTA; IP, interpeduncular nucleus; M2, secondary frontal cortex; mlf, medial longitudinal fasciculus; MO, medial orbital cortex; ob, olfactory bulb; mp, mammillary peduncle; PAG, periaqueductal gray; PN, paranigral subdivision of VTA; ri, rhinal incisure; RLi, rostral linear raphe nucleus; RN, red nucleus; rs, rhinal sulcus; SC, superior colliculus; SNc, substantia nigra pars compacta; SNr, substantia nigra pars reticulata;. Scale bars, **B**, 1 mm, **E-F**, 500 μm, **G-H**, 100 μm.

The animals were classified in three groups according to the mPFC injection sites. The first group comprised ten animals with Fluorogold (FG) injections located exclusively in the prelimbic sector (PL) of mPFC (Figure 1A,B). In another eight animals, the FG deposit also included either the medial orbital sector (MO; n = 4) or the cingular sector (Cg1; n = 4) of mPFC. In sixteen animals, the retrograde tracer stained all layers of the cerebral cortex; in one animal (R31; PL) the FG deposit was mainly in superficial layers (layers I - IV; PL) and in another animal (R40; Cg1-PL) the FG injection was confined to layers I-V (Cg1-PL).

### Single immunochemical detection of Fluorogold

All the animals had FG-immunolabeled neurons in VTA, but these neurons were also observed in some other brainstem regions such as the locus coeruleus, dorsal raphe nucleus and laterodorsal tegmental nucleus. A few FG-immunoreactive neurons were also found in the pedunculopontine nucleus, lateral parabrachial nucleus, caudal linear nucleus and median and pontine raphe nuclei (Figure 1C-F).

FG-immunoreactivity was observed in cell bodies and dendrites of VTA multipolar neurons (Figure 1G,H). The FG-immunolabeled neurons were largely localized in the VTA ipsilateral to the injection site (n = 18; 87.14% ± 1.57%; Figure 1E,F,G; 2A). The ipsilateral versus contralateral distribution of FG retrogradely-labeled neurons was quite alike in the three injection groups (Table 1), and FG-labeled neurons were significantly more frequent in ipsilateral than contralateral VTA in the three injection site groups (PL: F_1,18_ = 524.29; p < 0.0001; Cg1-PL F_1,6_ = 2363.40; p < 0.0001; PL-MO F_1,6_ = 556.97; p < 0.0001; Table 1). In most of the animals (n = 15), FG-immunolabeled neurons were evident throughout the entire rostro-caudal extension of the VTA, although in the remaining three animals (R31, PL; R27 and R37, PL-MO) they were only observed in rostral VTA. Moreover, retrograde-labeled neurons were more frequent in the parabrachial subdivision (PBP; 61.96% ± 2.11%; Figure 1E-H) than in the paranigral subdivision (PN) of the VTA (26.66% ± 2.32%). Lower proportions of FG-labeled neurons were observed in the interfascicular subdivision (IF) of the VTA (6.79% ± 1.43%) and in rostral and caudal linear raphe nuclei (4.59% ± 1.29%). FG-immunoreactive neurons were more densely grouped in medial and ventral portions of VTA. This distribution pattern for FG neurons in the different VTA subnuclei was independent of the injection site (F_6,60_ = 63.53; p = 1.02).

**Table 1:** **Fluorogold-labeled neurons in the ventral tegmental area by either immunofluorescence or immunohistochemistry**

		PL (%)	PL-MO (%)	Cg1-PL (%)
IH	Ipsilateral	84.18 ± 2.11	93.63 ± 2.61	88.06 ± 1,11
	contralateral	15.82 ± 2.11	6.37 ± 2.61	11.94 ± 1.11
IF	Ipsilateral	85.60 ± 4.77	90.47 ± 3.17	85.56 ± 6.93
	contralateral	14.40 ± 4.77	9.53 ± 3.17	14.44 ± 6.93

Percentage of Fluorogold (FG)-immunoreactive neurons (Mean ± SE) within the ipsilateral and the contralateral ventral tegmental area to the FG injection site in the three injection site groups using immunohistochemistry (IH) or immunofluorescence (IF) techniques. Cg1: cingular cortex (n = 4 animals); MO: medial orbital cortex (n = 4 animals); PL: prelimbic cortex (n = 10 animals). Percentages were calculated from the FG-immunolabeled neurons counted in 8 vibratome sections per animal in IH experiments and in 5 vibratome sections per animal in IF experiments.

### Dual immunofluorescent detection of Fluorogold and Tyrosine Hydroxylase

**Figure 2:**
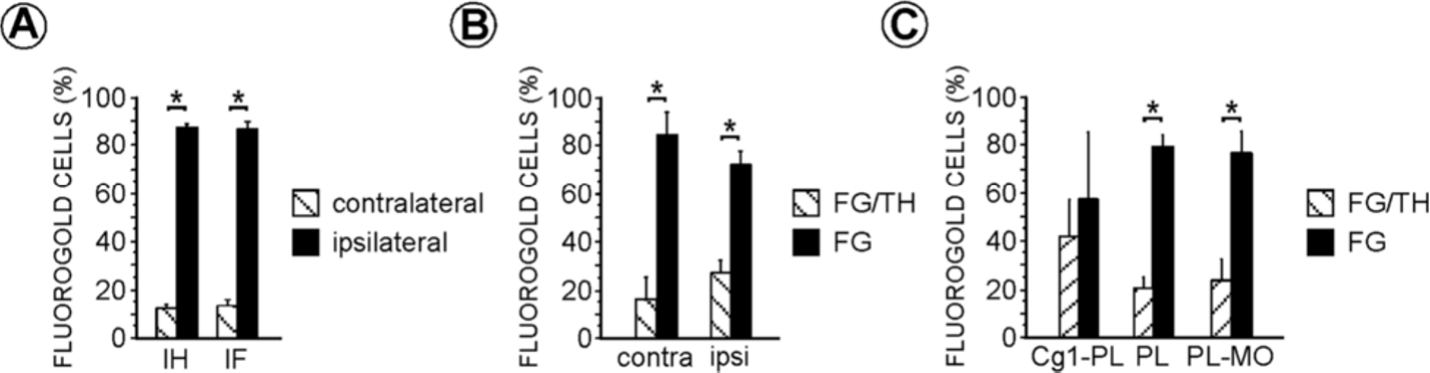
**Bar graphs summarizing the percentage distribution of Fluorogold (FG) neurons obtained with the different types of immunolabeling techniques in the ipsilateral and the contralateral ventral tegmental area to the FG injection site in either PL, Cg1-PL or PL-MO sectors of the medial prefrontal cortex. (A)** Bar graph showing the percentage distribution of FG-immunolabeled neurons detected with either DAB-immunohistochemistry (IH) or immunofluorescence (IF) methods in the ventral tegmental area ipsilateral and contralateral to the FG injection site in the medial prefrontal cortex. Percentages (mean ± SE) were calculated based on the numbers (961 FG-labeled neurons) obtained from 144 vibratome sections in 18 rats. ANOVA (animal X immunolabeling method X labeling side) was used for statistical comparisons [*p < 0.0001; post hoc Fisher test, factor: labeling side]. **(B)** Bar graph showing the proportions of single FG- and double FG/tyrosine hydroxylase (TH)-immunolabeled neurons observed in either ipsilateral (ipsi) or contralateral (contra) VTA in tissue double processed for FG and TH. ANOVA (animal X labeling side X single or dual labeling) was used for statistical comparisons [*p < 0.0001; post hoc Fisher test, factor: single or dual labeling]. **(C)** Bar graph showing the percentage of single FG- and double FG/TH-immunolabeled neurons observed in the three injection groups. Cg1, anterior cingulate cortex; MO, medial orbital cortex; PL, prelimbic cortex. ANOVA (animal X injection site X single or dual labeling) was used for statistical comparisons [*p < 0.0001; post hoc Fisher test, factor: single or dual labeling]. In **B** and **C** percentages (mean ± SE) were calculated based on the numbers (466 FG-labeled neurons) obtained from 90 vibratome sections in 18 rats.

The distribution and morphology of the FG-immunofluorescent neurons in mPFC-injected animals were very similar to those described above observed using the immunoperoxidase technique (n = 18; ipsilateral 86.69% ± 3.04%; contralateral 13.31% ± 3.04%; Figure 2A), and did not differ significantly with injection site (PL, PL-MO, Cg1-PL; F_2,30_ = 0.416; p = 0.66, Table 1). The location of FG-labeled neurons in the different subdivisions of VTA was similar to the location observed using immunohistochemical staining (χ^2^_3_ = 2.57; p = 0.46).

**Figure 3:**
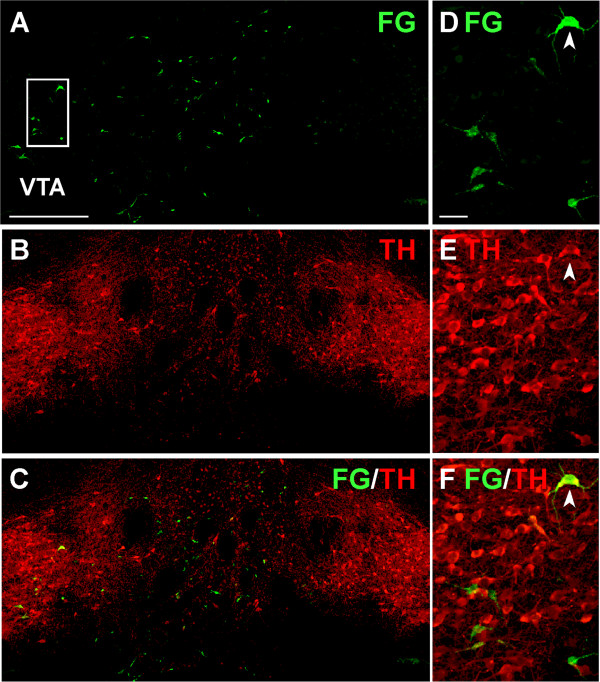
**Confocal photomicrographs showing Fluorogold (FG)- and/or tyrosine hydroxylase (TH)-labeled neurons in the ventral tegmental area (VTA) of an animal with an FG injection in the prelimbic cortex. (A-C)** *,* Biomappings showing green FG-labeling **(A)**, red TH-labeling **(B)** and both (FG/TH) labelings (orange) in the merged image **(C). (D-F)** *,* High power confocal photomicrographs of the boxed area in **A** corresponding to the VTA ipsilateral to the FG injection, depicting FG **(D)**, TH **(E)** and their merged images **(F)**. Arrowhead marks one VTA neuron dually labeled for FG and TH. Scale bar, **A-C**, 400 μm; **D-F**, 40 μm.

In the VTA, less than 30% of the FG-immunoreactive neurons also contained TH (n = 18; 26.05% ± 5.65%; Figures 2B, and 3); this proportion was similar in both the ipsilateral and contralateral VTA (Figure 2B). ANOVA showed that FG/TH-immunolabeled neurons were statistically less numerous than single FG-labeled neurons (F_1,34_ = 35.91; p < 0.0001) and this was observed in animals after injections in PL (F_1,18_ = 72.27; p < 0.0001) and PL-MO group (F_1,6_ = 16.90; p = 0.006) but not in the Cg1-PL group (F_1,6_ = 0.29; p = 0.61; Figure 2C). Three animals (PL, R24, R36; Cg1-PL, R30) did not have FG-labeled neurons in the VTA contralateral to the injection site.

### Single immunochemical detection of Hcrt1/OxA

**Figure 4:**
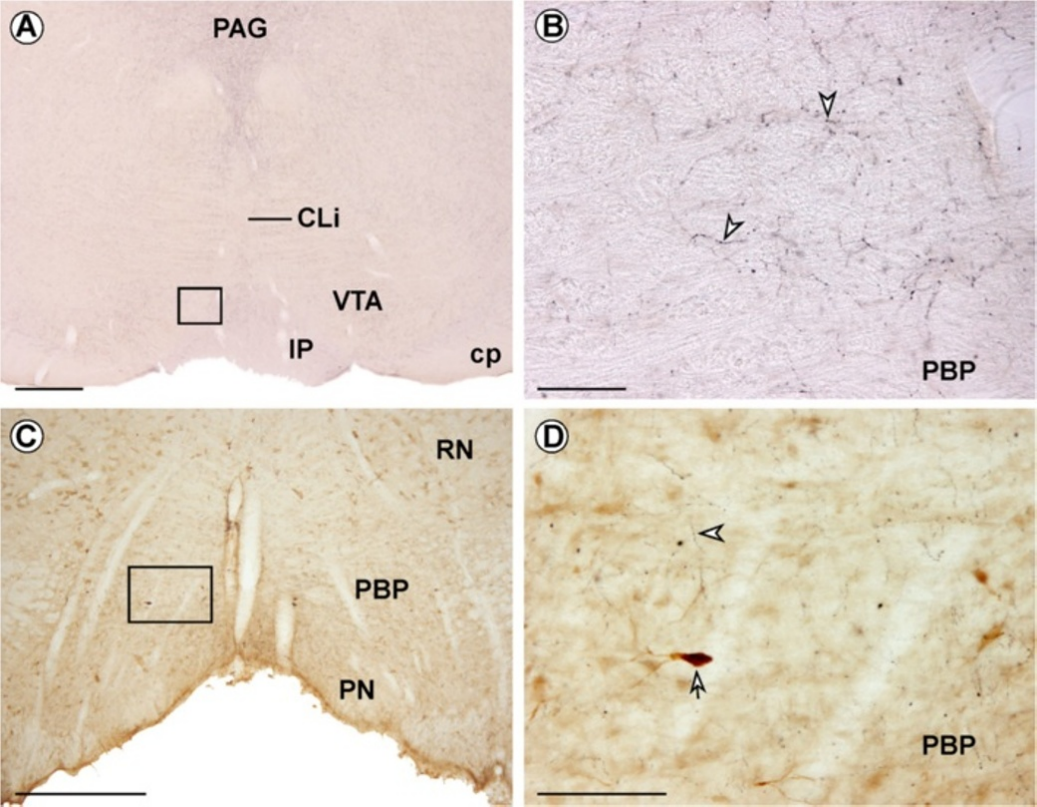
**Hcrt1/OxA immunoreactivity in the ventral tegmental area (VTA). (A) Coronal section showing Hcrt1/OxA-immunoreactive fibers in the VTA. (B)** High magnification of boxed area in A illustrating Hcrt1/OxA-containing axons. Arrowheads point to Hcrt1/OxA varicose axons in the parabrachial subdivision of VTA. **(C)** Double immunolabeling for Fluorogold (DAB, brown immunoprecipitate) and Hcrt1/OxA (DAB-nickel, black immunoprecipitate) in the VTA. **(D)** High magnification of boxed area in **C**, showing an Hcrt1/OxA containing axon (arrowhead) close to a Fluorogold-labeled neuron (arrow) within the parabrachial subdivision of VTA ipsilateral to the injection site. IP, interpeduncular nucleus; PAG, periaqueductal gray; PN, paranigral subdivision of the VTA; CLi, caudal linear raphe nucleus; cp, cerebral peduncle; RN, red nucleus. Scale bar, **A,C**, 500 μm; **B,D**, 100 μm.

Midbrain sections from four rats without an FG injection in mPFC were immunolabeled for Hcrt1/OxA. Hcrt1/OxA-immunoreactive axons were observed throughout the whole rostro-caudal and dorso-ventral extents of the VTA, as has been previously described [10, 11]. Hcrt1/OxA containing axons were scattered enough to make it possible to distinguish their varicosities and axon trail quite easily (Figure 4A,B). The diameter of Hcrt1/OxA-immunoreactive varicosities was 0.351 ± 0.103 μm, (mean ± standard error) as measured with ImageJ software at 100× magnification (n = 582), whereas intervaricose Hcrt1/OxA-immunoreactive segments were always less than 0.236 μm wide.

### Dual immunohistochemical detection of Fluorogold and Hcrt1/OxA

Observation of the tissue sections dually labeled for FG and Hcrt1/OxA only showed that a very few FG retrograde-labeled neurons and Hcrt1/OxA containing varicosities were apposed to each other in the same focal plane at light microscope level (Figure 4C,D), suggesting that cellular contacts might be unusual between FG-immunoreactive neurons and Hcrt1/OxA-labeled axons in the VTA.

**Figure 5:**
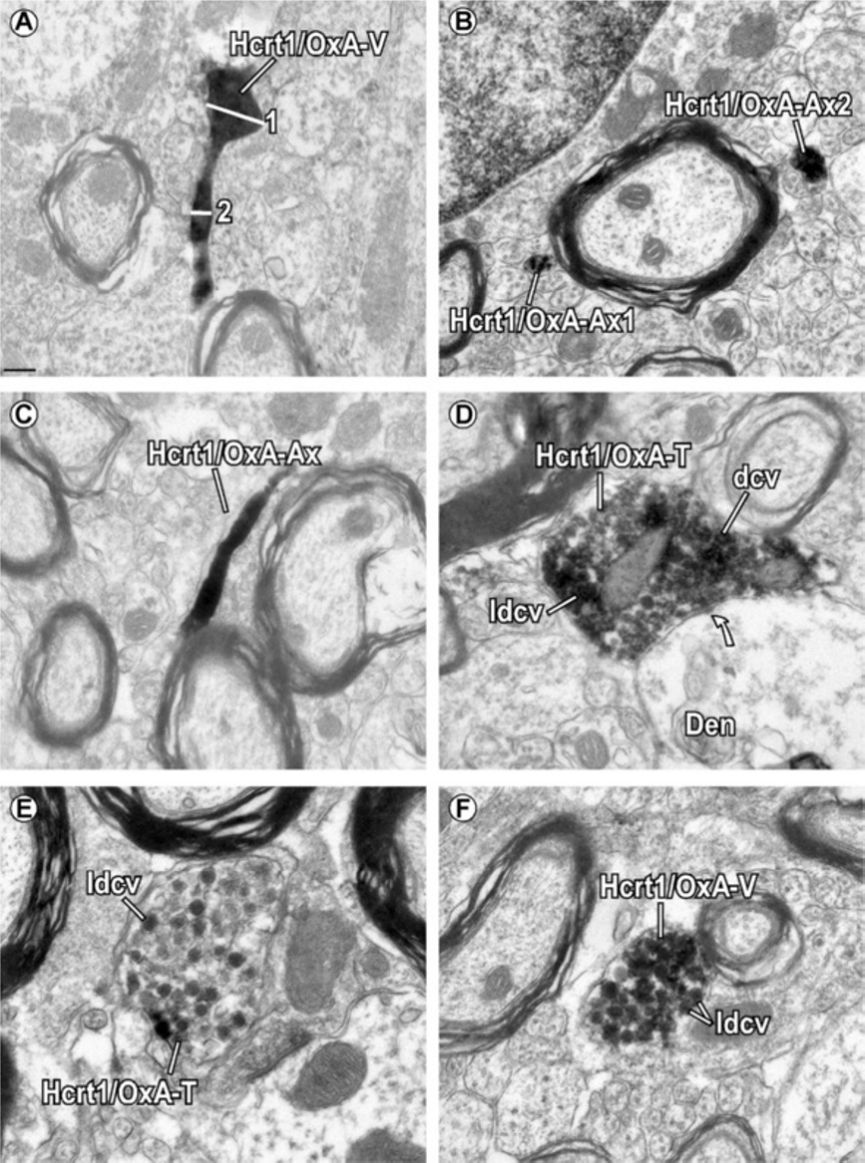
**Subcellular location of Hcrt1/OxA-immunoreactivity in axonal profiles of the ventral tegmental area. (A)** Hcrt1/OxA location in an axon sectioned so that the same image contains a preterminal varicose enlargement (Hcrt1/OxA-V; 1; diameter = 0.347 μm) connected to a short narrowing of the axon (2; diameter = 0.125 μm). **(B)** Hcrt1/OxA-immunolabeled transverse-sectioned unmyelinated axons (Hcrt1/OxA-Ax). Note that one of the axons only shows the Hcrt1/OxA reaction product in some parts of its cytoplasm (Hcrt1/OxA-Ax1), while the other (Hcrt1/OxA-Ax2) is completely filled by DAB-nickel-immunoprecipitate. **(C)** Longitudinally-sectioned Hcrt1/OxA unmyelinated axon. **(D)** Hcrt1/OxA-immunolabelled axon terminal (Hcrt1/OxA-T) that makes an asymmetric synapse (curved arrow) on an unlabelled dendrite (Den). Note the presence of Hcrt1/OxA-immunoreaction product in dense-cored vesicles (dcv), large dcv (ldcv) and in specific regions of the cytoplasm. **(E)** Hcrt1/OxA-immunoreactivity is observed exclusively within large dense-cored vesicles (ldcv) of an axon terminal (Hcrt1/OxA-T). **(F)** Varicosity showing a dense DAB-nickel precipitate for Hcrt1/OxA in ldcv and the cytoplasm. Scale bar, **A-C**, **E-F**,0.2 μm; D, 0.5 μm.

Six animals with PL injections were analyzed for dual FG- and Hcrt1/OxA-labeling with the electron microscope. FG peroxidase immunoreaction product was observed in the cytoplasm, multivesicular bodies and lysosomes of cell bodies and dendrites. Hcrt1/OxA was only found in axonal profiles. Hcrt1/OxA-immunoprecipitate was observed in the cytoplasm, in dense-cored vesicles (dcv) and in large dense-cored vesicles (ldcv), usually filling the profiles completely (Figure 5A-C). However, in some axons Hcrt1/OxA was exclusively observed in vesicles and/or discrete portions of the cytoplasm (Figure 5B,D-F). Hcrt1/OxA-containing axons were detected throughout the whole thickness of the VTA sections, indicating optimum antiserum penetration. There were no significant variations in Hcrt1/OxA-immunoreactive axon area density (number of labeled profiles per square micrometer of analyzed surface) between animals (F_5,50_ = 2.36; p = 0.06) or ultrathin sections (F_13,42_ = 0.93; p = 0.53). These findings demonstrate that Hcrt1/OxA-immunoreactivity in our sample was quite homogeneous. Most axonal profiles that contained Hcrt1/OxA were unmyelinated axons (diameter ≤ 0.235 μm; n = 1558; 67.42% ± 4.75%; Figure 5B,C) and some profiles were varicosities (diameter 0.236-0.699 μm; n = 579; 29.76% ± 4.26%; Figure 5A,F). There were also a few axon terminals (diameter ≥ 0.700 μm; n = 37; 1.71% ± 0.50%; Figure 5D,E). Three animals (R35, R36, R38) showed a few Hcrt1/OxA myelinated axons (n = 12; 1.11% ± 0.75%). The distribution of the different axonal profile types (myelinated, unmyelinated, varicosity and terminal) in a sample that contained all the profile types of Hcrt1/OxA axons and in another sample containing only axons with a circularity ≥ 0.7 was not significantly different (χ^2^_3_ = 0.64; p = 0.89). This shows that the criterion used for measuring axon diameter was a good estimate of their real diameter, thus validating the utilization of the whole axonal sample.

Unmyelinated Hcrt1/OxA-immunolabeled axons were mainly sectioned on a transverse plane (76.84% ± 1.83%; Figure 5B), but they were also sometimes observed in non-transverse or longitudinal cross-sectioned planes (Figure 5C). The ANOVA test showed that there were no significant variations between animals regarding the preferential transverse sectioning of Hcrt1/OxA axons (F_5,16_ = 0.40; p = 0.84) (data not shown). Longitudinally-sectioned unmyelinated Hcrt1/OxA-labeled axons frequently showed dcv and ldcv. Most Hcrt1/OxA-labeled axons were grouped with other axons in bundles within the neuropil and did not make contacts with VTA profiles, suggesting that they would mainly be *en passant* fibers traversing the VTA towards other brain regions. Hcrt1/OxA-immunolabeled axonal boutons were also mostly cut transversely (94.85% ± 1.07%) in our ultrathin sections, and there were no statistical differences between animals in the preferential transverse sectioning of Hcrt1/OxA containing boutons (F_5,16_ = 1.22; p = 0.34) (data not shown). The varicosities were the main Hcrt1/OxA-immunoreactive axonal bouton type (94.58% ± 1.47%), and their percentage was fairly uniform in all the animals (F_5,16_ = 1.65; p = 0.20).

**Figure 6:**
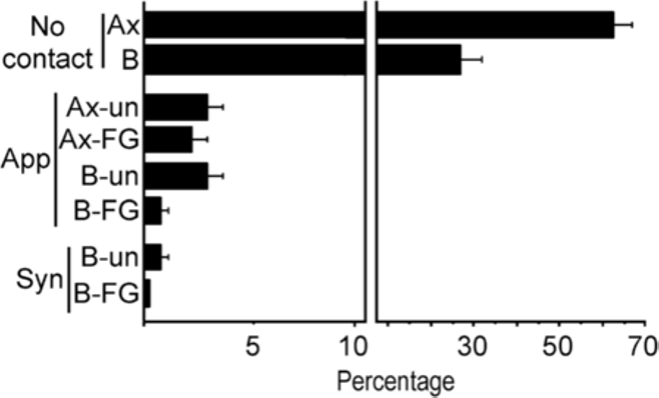
**Bar graph showing the percentage distribution of contact types (apposition [App] or asymmetric synapse [Syn]) or absence of contact (No cont) between Hcrt1/OxA-containing unmyelinated axons (Ax) or boutons (B) with ventral tegmental area dendrites that are unlabeled (un) or Fluorogold-labeled (FG).** Percentages (mean ± SE) were obtained from 14 vibratome sections of 6 rats, representing the distribution of a total number of 2,174 Hcrt1/OxA-immunolabeled axons with respect to their contacts with dendrites within the ventral tegmental area.

**Figure 7:**
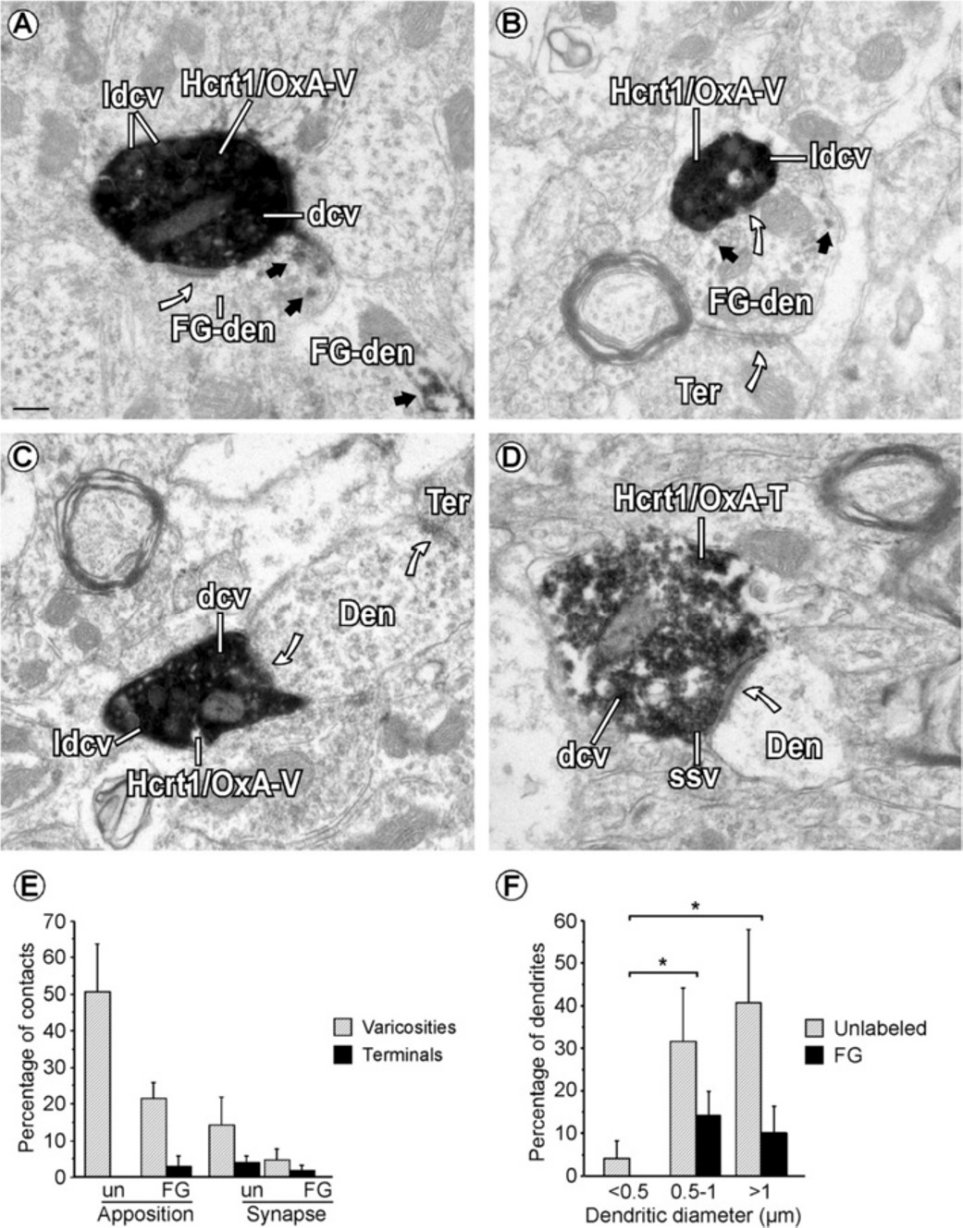
**Cellular contacts established by Hcrt1/OxA-containing axons in the ventral tegmental area. (A)** Hcrt1/OxA-immunoreactivity is seen in large dense-cored vesicles (ldcv), dense-cored vesicles (dcv) and in the cytoplasm of a varicosity (Hcrt1/OxA-V) that makes an asymmetric synapse (curved arrow) with a Fluorogold-labeled dendrite (FG-den). The FG-den is identified by its content in DAB-immunoperoxidase reaction product (black arrows). **(B)** Hcrt1/OxA-V establishes an asymmetric synapse (curved arrow) with a FG-dendrite that receives convergent input from an unlabeled axon terminal (Ter). **(C)** A VTA dendrite (Den) receives a synaptic contact (curved arrow) from an Hcrt1/OxA-immunoreactive varicosity (Hcrt1/OxA-V) and an unlabeled Ter. **(D)** Hcrt1/OxA-T makes an asymmetric synapse (curved arrow) on an unlabeled Den. Hcrt1/OxA-T contains translucent small synaptic vesicles (ssv) near the synaptic specialization while dcv are far from the synapse. **(E)** Bar graph showing the relative percentage of appositional and synaptic contacts (asymmetric) established by Hcrt1/OxA-boutons with unlabeled- (un) or FG-labeled (FG) dendrites according to the type of axonal bouton (varicosity, diameter < 0.7 μm or axon terminal, diameter ≥ 0.7 μm) in the ventral tegmental area. Mean percentages and standard errors were calculated based on the numbers obtained from 102 Hcrt1/OxA-immunoreactive boutons in 14 vibratome sections from six rat brains. **(F)** Bar graph showing the percentage distribution of unlabeled and Fluorogold-labeled (FG) dendrites of different sizes receiving asymmetric synapses (n = 22) from Hcrt1/OxA-immunolabeled axonal boutons (terminals and varicosities; total sample: 616 boutons) in the ventral tegmental area. Mean percentages and standard errors were calculated based on the numbers obtained from the synapse-recipient 22 dendrites in 18 ultrathin sections from 6 rat brains. ANOVA (animal X dendritic size) was done to determine in those dendrites significant variations in the formation of asymmetric synapses with respect to their small (<0.5 mm), intermediate (0.5-1.0 mm) or large (>1.0 mm) diameters [*p < 0.05; post hoc Fisher test]. Scale bar, 0.2 μm.

A small percentage of Hcrt1/OxA-containing axons established cellular contacts (appositions and synapses) with cellular bodies or dendrites of VTA neurons (11.20% ± 1.31%; Figure 6; Table 2). Hcrt1/OxA-immunoreactive axonal boutons preferentially made appositional contacts with VTA dendrites (n = 80; Table 2), most of which were unlabeled. In those cases in which synaptic specializations were evident, they were asymmetric (excitatory type; n = 22; Figure 7), and mainly formed with unlabeled dendrites (68.18%; Figure 7C,D), but contacts were also established with FG-labeled dendrites (31.82%; Figure 7A,B). Moreover, we observed one axo-somatic and one axo-axonic synapse with unlabeled profiles.

**Table 2:** **Appositional contacts and synapses established by Hcrt1/OxA profiles in the ventral tegmental area**

Hcrt1/OxA axonal profile type	Appositional contacts		Synapses (asymmetric)		Total
	FG	Unlabeled	FG	Unlabeled	
Unmyelinated axon (n = 1558)	42	96	2	5	145
Varicosity (n = 579)	20	55	4	11	90
Axon terminal (n = 37)	5	0	3	4	12
Total	67	151	9	20	247

Raw numbers of appositional contacts and synapses made by Hcrt1/OxA axonal profiles in the ventral tegmental area with either Fluorogold (FG)-labeled or unlabeled dendrites. Data were collected from 14 vibratome sections processed for dual labeling (Hcrt1/OxA and FG) in 6 rats that had been injected with FG in the prelimbic sector of the medial prefrontal cortex.

The presence of cellular contacts made by Hcrt1/OxA-immunolabeled boutons was frequently associated with a given morphological type of axonal bouton (χ^2^_1_ = 7.18; p = 0.007). Hcrt1/OxA-immunoreactive axon terminals established about twice as many cellular contacts (apposition and synapse) as Hcrt1/OxA-immunolabeled varicosities. Furthermore, analysis of the synaptic proportions with respect to all cellular contacts made by both types of bouton (varicosity or axon terminal), showed that Hcrt1/OxA-labeled axon terminals made more synapses than varicosities (58.33% versus 16.67% of total contacts). Thus, Hcrt1/OxA axon terminals preferentially formed asymmetric synapses while Hcrt1/OxA varicosities mainly established appositional contacts (Figure 7E; Table 2). This association between axonal bouton type (varicosity or terminal) and cellular contact type (apposition or synapse) was assessed using the chi-square test for the raw number of contacts made by Hcrt1/OxA-immunoreactive boutons (χ^2^_1_ = 10.87; p = 0.001; Table 2). Most of the dendrites that established asymmetric synapses with Hcrt1/OxA-labeled boutons had a mean diameter greater than 0.8 μm (62.50%; Figure 7F), suggesting they were close to the neuronal somata. We only observed one Hcrt1/OxA varicosity that formed a synapse with an unlabeled dendrite that had a diameter less than 0.5 μm. Some dendrites that established synapses with Hcrt1/OxA boutons also received inputs from unlabeled axon terminals that made cellular contacts (appositions or synapses) with FG-labeled dendrites.

**Figure 8:**
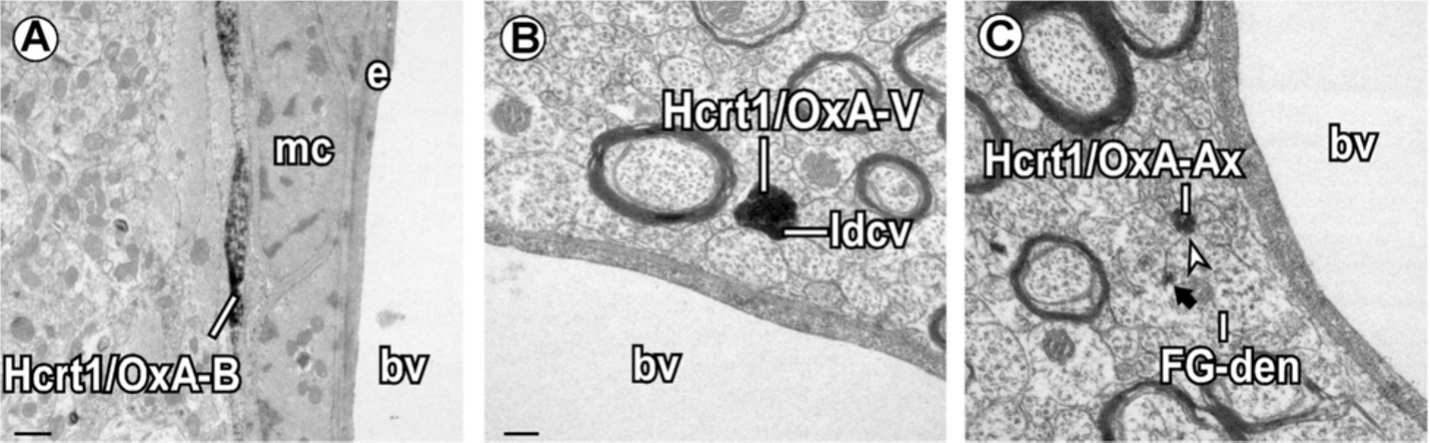
**Hcrt1/OxA-containing axons close to blood vessels (bv) in the ventral tegmental area. (A)** An Hcrt1/OxA-immunoreactive axonal bouton (Hcrt1/OxA-B) forms an appositional contact with a muscle cell (mc) in an arteriole. **(B)** Hypocretinergic/orexinergic varicosity (Hcrt1/OxA-V), containing large dense-cored vesicles (ldcv), is near a bv. **(C)** Unmyelinated axon containing Hcrt1/OxA (Hcrt1/OxA-Ax) close to a bv, makes an appositional contact (arrowhead) with a Fluorogold-labeled dendrite (FG-den, black arrow). e, endothelial cell. Scale bar, **A**, 0.5 μm; **B-C** *,* 0.2 μm.

A few Hcrt1/OxA-containing unmyelinated axons, most of them transversely-sectioned, formed appositional contacts (138/1559; Figure 7A) with VTA dendrites [unlabeled (3.59% ± 0.88%); FG-labeled (2.19% ± 0.46%)]. In only two rats (R31 and R35) did these axons make some asymmetric synapses onto dendrites [unlabeled (5/1558); FG-labeled (2/1558); Table 2]. Furthermore, Hcrt1/OxA-containing axons were close to blood vessels or even in contact with the vascular glia limitans (Figure 8). In our sample, around 2% of the Hcrt1/OxA-immunoreactive axons were located at a distance of less than 0.6 μm from the vascular basal membrane (1.99% ± 0.42%); approximately half of these axons close to blood vessels were boutons (1.07% ± 0.60%).

## Discussion

The Hcrt/Ox system sustains arousal [26] and supports the transition of sleep-wakefulness cycle states [27]. In the present study, we suggest that some of these Hcrt/Ox functions could be mediated by their actions on VTA neurons (dopaminergic and non-dopaminergic) that project to the mPFC. Furthermore, we demonstrate that non-dopaminergic neurons constitute a major source of the mesocortical pathway to mPFC.

### Methodological considerations

The tracer FG was used because it can be retrogradely transported to neuronal somata that are a long distance from the tracer deposit, and it can be detected by different immunohistochemical techniques that amplify the FG signal [28, 29]. However, we cannot exclude the possibility that some axon terminals did not take up the FG and the retrogradely-labeled neurons would be underestimated. Injection parameters such as current intensity, duration of injection and micropipette tip diameter were the optimal to obtain small injections that would include all cortical layers without producing cortical lesions. All antisera used have a high specificity and have been used in previous studies [28, 30, 31]. Moreover, omission of the primary antibodies resulted in the absence of immunoreactivity. The Hcrt1/OxA antiserum has been characterized at light and electron microscope levels and it does not cross-react with Hcrt2/OxB antisera [30]. What is more, Hcrt1/OxA-immunolabeling was never observed in somatodendritic profiles, but only in axons in the VTA, as we would have expected, whereas FG is mainly associated with lysosomes and multivesicular bodies of proximal dendrites and somata. FG-immunoprecipitate was also observed in confined zones of the cytoplasm. Hcrt1/OxA detection was amplified using immunoperoxidase-nickel-intensification, and the intense electrodense labeling was easily discernible from FG-immunoprecipitate. Overall, the immunohistochemical procedures employed allowed the identification of specific labeling in material in which there was almost no background, as judged by stray immunoprecipitate over resin, myelin, or other structures not expected to express the antigens they were raised against.

Although Hcrt1/OxA-containing axons in our sample only made a few synaptic contacts, we should recall that synaptic specializations occupy a small proportion of the neuron membrane surface and we did not study serial ultrathin sections. Thus, we cannot exclude the possibility that Hcrt1/OxA-immunolabeled axons might have formed synaptic contacts with FG-labeled neurons in sectional planes other than the ones observed here. Moreover, pre-embedding methods can underestimate the associations between immunoreactive profiles [32]. In addition, since we injected only a small cortical area (PL), but VTA has a widely distributed projection system to cerebral cortex [4], the observed synaptic contacts with FG-dendrites are only a fraction of the total synapses onto mesocortical neurons. However, they represent most of the synaptic contacts with VTA neurons that project to mPFC, since our injections in all cases comprised the whole mPFC. Moreover, FG has proved to have a good efficiency as a tracer for VTA projections to mPFC in numerous studies [6, 28]. The high sensitivity of FG as retrograde tracer [29, 33] suggests that most mesocortical neurons reaching the mPFC were detected in our animals. There is no reason to suppose that Hcrt1/OxA axons innervate preferentially a neuronal population that had not transported the FG, resulting in an underestimation of contacts.

### Mesocortical system: the relative contribution of dopaminergic neurons

It is known that many cognitive and attentional processes characteristic of wakefulness are mediated by VTA projections to mPFC [34]. Our FG-labeled neurons were mainly located in the ipsilateral parabrachial division of the VTA as reported in similar studies previously done in rats [5, 35, 36] and monkeys [37], even though in some of those studies the injections were more posterior and included all the anterior cingular cortex [5]. More recent studies have described that PL injections mainly label neurons within the parabrachial VTA of the rat [28], but some others have reported to label equal numbers of paranigral and parabrachial neurons after injections in posterior areas of anterior cingular and prelimbic cortices in mice [38].

Our finding of many non-DA VTA neurons projecting to the mPFC agrees with previous results in rats from Swanson (1982) [5], Carr & Sesack [6] and Lammel *et al*., [38]. In addition to DA neurons, glutamate and GABA neurons are present in the VTA [2, 3], but the distribution of these neuronal populations in the different subdivisions of the VTA is heterogeneous. Although PBP contains many DA neurons [2], the VTA PBP neurons projecting to PFC observed in this study and another [5] are mainly non-dopaminergic. Moreover, subcellular studies have determined that around 60% of VTA neurons projecting to mPFC contain GABA [6]. Furthermore, Lammel *et al*., [38] have also described that DA neurons projecting to PFC were located mainly in the PN and caudal PBP subdivisions of VTA, while neurons in the rostral PBP subdivision that project to mPFC were mostly non-DA, as occurred in our animals.

VTA actions on the frontal cortex are mediated by both DA and non-DA neurons. Since non-DA mediated cortical activity can be blocked with GABA antagonists, these mesocortical neurons are presumed to be mainly GABAergic [8]. Moreover, electrophysiological unit recordings combined with immunohistochemical detection have demonstrated that many VTA mesocortical neurons supposedly involved in reward behaviors and cortical arousal [39] are indeed GABAergic [40]. However, we cannot discard that non-DA mesoprefrontal neurons could contain some other neurotransmitters such as glutamate, whose presence in VTA neurons is unequivocally reported [3]. There is also evidence that DA VTA neurons are unique in their firing rate stability across sleep states [41]. On the contrary, VTA GABA neurons increase firing rates during active wakefulness and REM sleep in comparison to quiet wakefulness and non-REM sleep [7]. This suggests that GABA VTA neurons could be more involved in extrathalamic cortical activation [7] while DA neurons seem to be implicated in responses to non-predicted rewards or salience [42].

### Hcrt1/OxA targeting of VTA mesocortical neurons

Previous studies on preproHcrt/preproOx [12] and Hcrt1/OxA [23] distributions have reported that Hcrt/Ox-containing axon density in VTA is weaker than in other nuclei such as the locus coeruleus or dorsal raphe nucleus, similarly to our results. Balcita-Pedicino & Sesack [23] observed that Hcrt1/OxA and Hcrt2/OxB had similar distributions and locations in VTA varicose axons. These authors described Hcrt/Ox-immunoreactivity as being located in the cytoplasm and dense-cored vesicles of different sizes (dense-cored vesicles, dcv and large dense-cored vesicles, ldcv) within axons. Furthermore, they reported that only 15% of the Hcrt1/OxA-containing axons made some cellular contact (apposition or synapse) with VTA neurons. In our study, the proportion of Hcrt1/OxA axons that established contacts is somewhat similar (11.20%) but, in contrast to their description of both asymmetric and symmetric synapses, we only observed Hcrt1/OxA axons making asymmetric synapses on VTA dendrites.

Hcrt1/OxA has been identified in dcv and ldcv previously [10, 43, 44]. These kinds of vesicles release their content by exocytosis [45, 46]. Torrealba *et al*., [43] have reported Hcrt1/OxA in ldcv and dcv within axons of the hypothalamic tuberomammillary nucleus. They observed in the same axon terminals that these vesicles were localized far from the synaptic specialization, while translucent small synaptic vesicles near synapses contained glutamate.

Hcrt1/OxA and Hcrt2/OxB activate both DA and non-DA VTA neurons [22]. In contrast to Hcrt1/OxA, Hcrt2/OxB has been reported to increase presynaptic glutamate release in addition to potentiation of postsynaptic NMDA receptors [47]. Furthermore, Hcrt/Ox enable glutamate-mediated responses in VTA that are necessary for glutamate-dependent long-term potentiation in VTA DA neurons [48]. Thus, Hcrt1/OxA and Hcrt2/OxB could modulate different behavioral components of VTA function.

FG injections in PFC combined with ventricular infusion of Hcrt1/OxA has shown that the neurons projecting to the PFC that are activated by Hcrt1/OxA are mainly located in caudal and medial portions of VTA, and that approximately 50% of them are dopaminergic [19]. In addition, local infusion of Hcrt1/OxA in VTA increases wakefulness and DA release in PFC [21].

The common observation of Hcrt1/OxA axons close to blood vessels, similar to what has been reported in other brain areas [49–51], suggests an association between the Hcrt/Ox system and vasomotor control. In fact, Hcrt/Ox-R1 activation attenuates neurogenic vasodilation of dural vessels [52]. Hcrt/Ox have also shown vasomotor effects through its activation of nitrergic or GABAergic neurotransmission in the solitary tract [53]. Neuropeptide Y, a well-known peptide involved in microvascular functions [54], is contained in hypothalamic neurons targeted by Hcrt/Ox axons [55]. Altogether, this suggests that the Hcrt/Ox system could be involved in vascular tone regulation also in the VTA.

### Functional considerations

Hcrt/Ox neurons have their highest firing rate in wakefulness, decrease activity during non-REM sleep, and are relatively silent in REM sleep [17]. Thus, Hcrt/Ox help in maintaining wakefulness [26] and mediate sleep-wakefulness transitions [27]. Hcrt1/OxA actions on the cortex are partly direct and partly due to activation of ascending systems reaching the cerebral cortex [56]. However, it is unknown, so far, which of these pathways is more relevant for arousal and wakefulness. Our results complement previous studies on Hcrt1/OxA innervation of brainstem wakefulness-related areas, [49–51], and allow us to establish cellular and subcellular bases by which the Hypocretinergic/orexinergic system excites VTA neurons that project to PL, activating the cerebral cortex. The VTA is not the only gateway for Hcrt1/OxA neurons to gain acces to the mPFC. Significant Hcrt1/OxA targeting has also been described in some other brainstem nuclei projecting to mPFC, such as the locus coeruleus [49], dorsal raphe nucleus [50] and laterodorsal tegmental nucleus [51]. Conceivably, all these structures contribute to cortical-activating actions of Hcrt1/OxA. The particular impact of the VTA within those actions seems to be especially prominent provided the strong dopaminergic regulation of both sleep attacks and cataplexy in murine and canine models of narcolepsy [57, 58]. The existence of Hcrt1/OxA-immunoreactive large dense-cored vesicles, which are quite distant from synaptic specializations in axons making asymmetric synapses, suggests that Hcrt1/OxA-containing axons may excite VTA neurons through both excitatory synapses and non-synaptic mechanisms, most likely through extracellular diffusion of the peptide, and activation of receptors away from the release site [24, 25]. Overall, this interpretation might help explain the hypersomnia of narcoleptic patients as the result in part of a reduced tone of Hcrt1/OxA arousing actions in the VTA [59].

**Figure 9:**
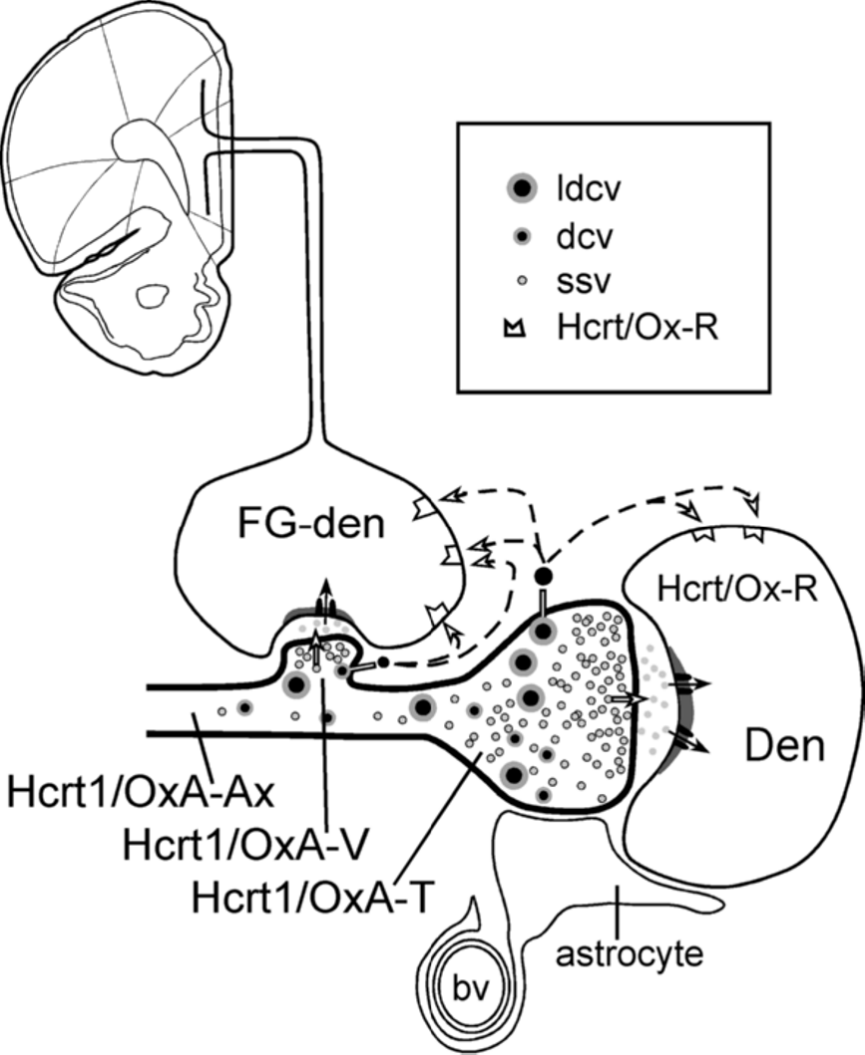
**Schematic drawing depicting the possible cellular mechanisms whereby Hcrt1/OxA activates neurons in the ventral tegmental area (VTA).** Hcrt1/OxA-containing axons (Hcrt1/OxA-Ax) make asymmetric synapses (excitatory type) with VTA dendrites (Den), some of which (Fluorogold-labeled dendrite, FG-den) belong to neurons that project to the medial prefrontal cortex. In the Hcrt1/OxA axons, translucent small synaptic vesicles (ssv) release their content to the synaptic cleft, activating postsynaptic receptors, while granular vesicles (dense-cored vesicles, dcv and large dcv, ldcv) may release Hcrt1/OxA far from synaptic specializations and activate extrasynaptic receptors (Hcrt/Ox-R) by volume transmission at a distance and more slowly. Both mechanisms (synaptic and non-synaptic) are presumably involved in Hcrt/Ox actions in the VTA, thus contributing to cortical activation and wakefulness maintenance. bv, blood vessel; Hcrt/Ox-R, receptor binding Hcrt1/OxA; Hcrt1/OxA-T, Hcrt1/OxA-containing axon terminal; Hcrt1/OxA-V, Hcrt1/OxA-containing varicosity.

The FG-labeled neurons observed in this study may be dopaminergic, GABAergic [6, 23] or glutamatergic [43]. The localization of Hcrt1/OxA-immunoprecipitate in axons forming asymmetric synapses and in granular vesicles (dcv and ldcv) that are distant from synaptic specializations, suggests that Hcrt1/OxA most likely colocalizes with an excitatory neurotransmitter such as glutamate in the VTA (Figure 9), similar to what has been described in the tuberomammillary nucleus [43]. Thus, Hcrt1/OxA could enhance the actions of an excitatory neurotransmitter during longer periods of time by rising excitability of common postsynaptic target neurons and/or temporal/spatial summation of individual actions of Hcrt1/OxA and the excitatory transmitter. Moreover, Hcrt/Ox inhibit brain structures involved in non-REM sleep generation, such as the preoptic area [20, 60, 61] and also blocks structures involved in REM sleep such as the ventral part of oral pontine tegmentum [62, 63].

## Conclusion

In the present study we demonstrate that Hcrt/Ox hypothalamic neurons may activate the medial prefrontal cortex through VTA neurons (dopaminergic and non-dopaminergic), contributing to the arousal and wake-enhancing functions typical of this cortical area. Hcrt1/OxA-containing axons may excite VTA neurons through both excitatory synapses and non-synaptic mechanisms (volume transmission). Both mechanisms (synaptic and volumetric) probably play important roles in cortical activation, enhancing the stabilization of the wakefulness-sleep states and maintaining arousal.

## Methods

### Fluorogold injections

Eighteen male adult Sprague–Dawley rats weighing 250-300 g were used in this study. All animal procedures were done in strict accordance to European Community Council Directive (86/609/EEC), and the used protocol was approved by the Ethical Committee for the use of laboratory animals of the Universidad Autónoma de Madrid. All efforts were made to minimize animal suffering.

To label VTA neurons projecting to mPFC, the retrograde tracer Fluorogold (FG; Fluorochrome, Englewood, CO) was microinjected into the prelimbic mPFC, extending in some cases to medial orbital and/or cingular sectors, using previously described methods [28]. The animals were anaesthetized with a cocktail of Ketamine (55 mg/kg i.m.), Xylacine (15 mg/kg i.m) and Atropine (0.2 mg/kg i.m.). Glass micropipettes were pulled to obtain 10-15 μm diameter tips and were filled with a 1.5% solution of FG in isotonic saline. The micropipettes were stereotaxically placed unilaterally in the mPFC (2.7 mm anterior to bregma; 0.4 mm lateral from midline; 3.7 mm ventral from skull dorsal surface), as determined from the rat brain atlas of Paxinos & Watson (1998) [64]. FG was injected iontophoretically using continuous current (positive 5 μA, on 8sg/ off 8sg) for 10–15 minutes. The micropipettes were left in place from 5 minutes before to 5 minutes after the injection to avoid spread of the solution. The skin incision was closed with a surgical thread and topical lidocaine was applied. The rats were returned to the animal colony and housed in individual cages. They were allowed unlimited access to water and food and were maintained in a 12 h light/12 h dark cycle for six days.

### Tissue preparation

Rats were perfused six days after FG injections, a time that has been shown optimal for retrograde tracing of VTA neurons after prefrontal cortex injections [33]. The rats were anaesthetized with sodium pentobarbital (33 mg/Kg i.p.) and their brains were fixed by aortic arch perfusion with heparin in saline (1000 U/ml) and 0.2% glutaraldehyde in a solution of 4% paraformaldehyde in 0.1 M phosphate buffer, pH 7.4 (PB). The brains were removed from the skull and coronal blocks containing the mPFC and the VTA region were obtained. They were postfixed for 2 hours in 4% paraformaldehyde in 0.1 M PB at 4°C, and coronal sections of 40-50 μm thickness were serially collected from these blocks in 0.1 M PB at 4°C, using a Vibratome Series 3000 (Technical Products International. Ted Pella. Inc).

One of the tissue series was stained using the Nissl method to locate the injections and the borders of the brain nuclei and tracts [64, 65]. The tissue sections used for immunohistochemistry were incubated for 15 minutes in 1% sodium borohydride in 0.1 M PB to inactivate free aldehydes and 15 minutes in 10% methanol and 10% hydrogen peroxide in 0.1 M PB to inactivate endogenous peroxidase. Then, the sections were processed for immunohistochemical labeling as described below. After the immunolabeling procedures, sections for light microscopy observation were rinsed in 0.03 M PB and mounted on gelatine-coated glass slides, dried, dehydrated in a series of ascending concentrations of ethanol, defatted in xylene and coverslipped with DPX mounting medium (Sigma-Aldrich, St. Louis, CA).

### Single immunohistochemical detection of Fluorogold

Sections containing either mPFC (15 sections per animal) or the VTA (8 sections per animal) region were processed immunohistochemically for light microscope observation of the injection site and retrograde tracing, respectively. They were first rinsed in 0.1 M PB and incubated 30 minutes in 0.5% bovine serum albumin (BSA; Sigma-Aldrich, St. Louis, CA) in 0.1 M PB to minimize non-specific background staining. They were then incubated overnight in rabbit anti-FG antibody (1:4000; AB153; Chemicon, Temecula, CA), 0.1% BSA and 0.25% Triton X-100 in 0.1 M PB protected from light at room temperature. After this, the sections were incubated in biotinylated goat anti-rabbit IgG (1:400; AP132B; Chemicon, Temecula, CA) and in 0.1% BSA in 0.1 M PB for 30 minutes, rinsed in 0.1 M PB and incubated 1 hour in avidin-biotin peroxidase complex (1:100; ABC; Vector Lab, Burlingame, CA). Peroxidase was visualized as a brown precipitate by incubation of the tissue in 0.022% 3,3´-diaminobenzidine (DAB) and 0.0033% hydrogen peroxide in 0.1 M PB at 4°C for 6–10 minutes.

### Dual immunofluorescence detection of Fluorogold and tyrosine hydroxylase

One of the tissue series was processed for immunofluorescent detection of the retrograde tracer FG and of tyrosine-hydroxylase (TH, marker for dopamine-containing neurons in the VTA). All procedures were done protecting the tissue from light. After extensive rinsing in 0.1 M phosphate-buffered saline pH 7.4 (PBS), the sections were incubated in citrate buffer pH 6.0 at 90°C for 10 minutes. Then the sections were incubated in 10% donkey serum, 1% BSA and 1% Triton X-100 in 0.1 M PBS for 2 hours at room temperature. Afterwards, tissue sections were incubated in 1) rabbit anti-FG (1:1500; AB153; Chemicon, Temecula, CA) and 2) mouse anti-TH (1:1000; 22941; ImmunoStar Inc, Hudson, WI) in 3% donkey serum, 0.3% BSA and 0.3% Triton X-100 in 0.1 M PBS at 4°C for 48 hours. Next, the sections were rinsed in 0.1 M PBS and incubated in donkey anti-rabbit IgG 488 nm (1:100; A21206; Alexa Fluor, Invitrogen, Carlsbad, CA) and donkey anti-mouse IgG 570 nm (1:100; 715-025-150; Jackson ImmunoResearch, West Grove, PA) in 0.1 M PBS at 4°C for 2 hours.

### Single immunohistochemical detection of Hypocretin1/OrexinA

Four additional naive male Sprague–Dawley adult rats weighing 250-300 g were used in these experiments. The Hcrt1/OxA antiserum used was raised in goat against a 19 residue-peptide fragment located at the C-terminus of human Hcrt1/OxA (aa 48–66 of the Hcrt/Ox precursor identical to the corresponding rat/mouse sequence). This antiserum has a highly specific distribution in multiple brain regions [17, 66]. Specificity of the anti-Hcrt1/OxA antiserum has been previously tested by Western blot and immunohistochemistry tests; the antiserum detects the processed active Hcrt1/OxA peptide of rat origin but it does not recognize or cross-react with Hcrt2/OxB [30]. Moreover, the specificity of the Hcrt1/OxA-labeling in the present study is also supported by the results of negative control experiments, and the species selectivity testing of the secondary antiserum.

Tissue sections were incubated in 10% donkey serum (Sigma-Aldrich, St. Louis, MO) in 0.1 M PB for 3 hours. They were then placed sequentially in: 1) goat anti-Hcrt1/OxA antiserum (1:2000; C-19: sc-8070; Santa Cruz Biotechnology Inc, St. Cruz, CA), 2% donkey serum and 0.25% Triton X-100 in 0.1 M PB at 4°C for 48 hours; 2) biotinylated donkey anti-goat IgG (1:400; AP180B; Chemicon, Temecula, CA) and 2% donkey serum in 0.1 M PB for one hour; and 3) ABC (1:100) in 0.1 M PB for an hour. Hcrt1/OxA was visualized as an intense black precipitate using a modification of the glucose oxidase-DAB-nickel method [67].

### Dual immunohistochemical detection of Hypocretin1/OrexinA and Fluorogold

Tissue sections of the VTA from the eighteen FG-injected rats were processed for dual detection of Hcrt1/OxA and FG. Non-specific tissue epitopes were blocked by incubation in 10% donkey serum in 0.1 M PB for 3 hours before incubating the sections in goat anti-Hcrt1/OxA (1:2000), 0.03% Triton X-100 and 2% donkey serum in 0.1 M PB at 4°C for 48 hours. Afterwards, the sections were incubated in biotinylated donkey anti-goat IgG (1:500) in 0.1 M PB for 2 hours, and in ABC (1:100) in 0.1 M PB for 1 hour. Hcrt1/OxA detection was completed by using the glucose oxidase-DAB-nickel method as described earlier. After abundant rinsing in 0.1 M acetate buffer pH 6.0 and in 0.1 M PB, the sections were sequentially incubated in 1) rabbit anti-FG antiserum (1:4000) and 2% donkey serum in 0.1 M PB at room temperature overnight; 2) unconjugated donkey anti-rabbit IgG (1:50; AP182; Chemicon, Temecula, CA) 2% donkey serum in 0.1 M PB for 30 minutes; and then, 3) rabbit peroxidase anti-peroxidase (1:500; PAP18; Chemicon, Temecula, CA) in 0.1 M PB for 2 hours. The peroxidase reaction product was visualized by incubation of the tissue in 0.022% DAB and 0.0033% hydrogen peroxide in 0.1 M PB for 10 minutes. The light brown immunoperoxidase reaction product identifying FG-labeled neurons was easily distinguishable from the nickel-intensified black reaction product that marked Hcrt1/OxA fibers.

### Electron microscopy

Six animals, which had FG injections restricted to the prelimbic cortex, were used for the ultrastructural study of dual Hcrt1/OxA and FG localization. Immunolabeled sections were postfixed in 2% osmium tetroxide in 0.1 M PB for an hour, dehydrated through a series of increasing ethanol concentrations and propylene oxide, and incubated overnight in a 1:1 mixture of propylene oxide and epoxy resin (Epon; EMbed-812; Electron Microscopy Sciences, Fort Washington, PA). The sections were transferred to 100% Epon for 2 hours, flat-embedded in Epon between two sheets of plastic film and cured at 60°C for 72 hours. Fragments of the VTA ipsilateral to the injection area at the approximate level of anteroposterior plane −5.3 mm from bregma were cut from the Epon-embedded sections and glued onto resin blocks. Ultrathin sections (40-50 nm) were obtained from the tissue-Epon interface using a diamond knife (Diatome MT 8618) within an ultramicrotome (Reichter-Jung, Ultracut E/GA/S-83/05; Vienna, Austria) and were collected on 300-mesh copper grids. Then, the ultrathin sections were counterstained with 5% uranyl acetate in water for 20 minutes followed by a solution containing 0.022 mg lead citrate in 0.5 ml NaOH and 4.5 ml distilled water for 4–6 minutes. They were examined with a Jeol JEM 1010 electron microscope (Tokyo, Japan) coupled to a Bioscan camera (Gatan Inc.).

### Data analysis

The tissue sections processed for single and dual-immunohistochemistry were studied with a Nikon Eclipse E600 microscope. Selected representative photographs of labeling for FG and/or Hcrt1/OxA were taken with the microscope equipped with a digital camera (digital camera DXM1200, Nikon) and appropriate computer software (Nikon Camera Software DXM1200 ACT-1). Adjacent Nissl sections were used to delimit FG deposits in mPFC (Figure. 1A) and to draw the retrograde-labeled neurons within the VTA (Bregma −5.3 mm to −6.8 mm). Outlines of sections and major brain structures were drawn by overlapping FG-immunolabeled sections and the adjacent Nissl-stained sections using a magnifying projector (Ernist leitz GMBH Wetzler; objective Milaron 1:2:5/90 mm); these drawings were scanned (EPSON Expression 1600) and FG-labeled neurons were plotted by using light microscope high magnification (20-40x). Two-way analyses of variance (ANOVA; labeling side X injection site group) were used to determine whether there was significant variability in the proportions of FG-immunolabeled neurons with respect to (1) ipsilateral versus contralateral side to the FG injection, and (2) location of injection site in mPFC (PL, Cg1-PL or PL-MO).

Immunofluorescence sections were examined by biomapping using a confocal microscope (Leica TCS SP2 Spectral microscope; Leica Microsystems, Germany); VTA area was studied in 1 μm thick optical sections obtained through the depth of the tissue with a 20X multi-immersion objective. FG single-labeled cells and FG/TH dual-labeled cells were counted throughout the VTA (n = 18). Analysis of variance (ANOVA) followed by Fisher’s least significant difference (PLSD) test were used to determine whether there were statistically significant variations in the percentage of these two neuronal populations between (1) immunolabeling side (ipsilateral versus contralateral to FG injection), and (2) different injection site groups (PL, Cg1-PL and PL-MO). Nested ANOVAs (labeling side X injection site X staining method) were used to assess the statistically significant variations in the proportions of single (FG) and dual (FG/TH) neurons in the three injection site groups (PL, Cg1-PL and PL-MO) using either immunohistochemistry or immunofluorescence. We also applied Chi-square test to determine the association among staining method and injection site group. The immunolabeled images and drawings were assembled and labeled with text in Canvas (Canvas X, ACD Systems International Inc., Canada) to obtain the composite figures depicting FG localization. Moreover, we determined the exact location of FG-labeled neurons in the different subdivisions of the VTA based on Halliday& Törk (1986) [1].

The ultrastructural analysis was carried out in 14 vibratome sections that were obtained from six rats (at least two sections per animal). Two ultrathin sections respectively cut from opposite sides of the vibratome sections (≥10 μm apart) were obtained in each animal from resin blocks including a VTA section. All immunoreactive processes (n = 2186) were counted in randomly sampled electron micrographs at 50,000x magnification from an area of 1,946,455.5 μm^2^ within an area of at least 90,859.96 μm^2^ examined in each animal. The classification of identified cellular elements was based on standard descriptions [68]. Axons were recognized by their small caliber and their lack of ribosomes. The maximum diameter along the short axonal axis was measured in Hcrt1/OxA-immunoreactive profiles as a criterion for estimating the real diameter of the profile regardless of the plane of section. The diameter is directly proportional to the cross-sectional surface of a profile, especially in rather cylindrical structures such as axons. We measured the circularity [*4π(area/perimeter*^*2*^*)*] in all the Hcrt1/OxA-containing axons. This parameter is a mathematical factor that indicates how close the diameter of a profile is to a perfect circle. Thus, circularities over 0.7 would indicate an axon was sectioned quite close to its transverse plane, while progressively lower circularities are found in axons that are longitudinally- or non-transversely-sectioned. Chi-square test (axonal profile type X circularity) was calculated to validate our method for measuring profile diameter regardless plane of section.

When sectioned transversely, axons had smooth contours and were often grouped in bundles. Longitudinally cut axons usually displayed varicosities with vesicles. Axon boutons were identified by the presence of numerous synaptic vesicles and were ≥ 0.236 μm in diameter. Varicosities (boutons *en passant*) ranged 0.236-0.699 μm in diameter and axon terminals were >0.7 μm, as measured both in random 100× micrographs in the light microscope images (ImageJ 1.40 software, National Institutes of Health, USA), and also in obviously longitudinally-sectioned varicose axons in electron microscope images. Thus, these criteria were used to classify an axonal profile as a varicosity or a terminal. Intervaricose segments of unmyelinated axons were < 0.236 μm wide and might or might not contain some vesicles. Dendrites usually contained abundant endoplasmic reticulum and were distinguished from unmyelinated axons by their larger diameter and/or prevalence of uniformly distributed microtubules. Neuronal somata were recognized by the presence of a nucleus, Golgi apparatus and rough endoplasmic reticulum. Both dendrites and somata were often found postsynaptically to axonal boutons. Synaptic contacts were classified as symmetric or asymmetric based on the characteristics of their pre- and postsynaptic densities. Zones of closely spaced parallel plasma membranes that lacked discernible synaptic densities, but were otherwise not separated by glial processes, were defined as appositions or non-synaptic contacts. All Hcrt1/OxA-immunoreactive axons within each ultrathin section were photographed at 50,000x with a digital camera coupled to the electron microscope and saved in TIFF format. The tissue was examined to determine: (1) the relative frequency with which Hcrt1/OxA immunoreactivity was located within axon boutons, unmyelinated axons or myelinated axons; (2) Hcrt1/OxA-immunolabeling area density (number of Hcrt1/OxA-immunolabeled profiles per analyzed surface unit) for each animal and ultrathin section; (3) the number and type of morphologically-recognizable synaptic or appositional contacts established by Hcrt1/OxA processes with FG-labeled or unlabeled profiles; and (4) measurements of area, perimeter, circularity and mean diameter of each Hcrt1/OxA-labeled profile (ImageJ). Analysis of variance (ANOVA) tests followed by *post hoc* Fisher´s PLSD post hoc test were used to determine whether there was significant variability in Hcrt1/OxA-immunolabeling area density with respect to different animals or sections. We also calculated ANOVAs (animal X axonal profile type X circularity) to assess significant statistical variations between animals in the distribution of transversely- and longitudinally-sectioned axons. Chi-square tests were used to determine the association among axonal profile type and the category of contact established (apposition or synapse). Significant statistical variations in the proportions of dendrites with different sizes receiving input from Hcrt1/OxA-immunolabeled boutons were evaluated with ANOVA (animal X dendritic diameter). Post hoc multiple pair analysis were made using Fisher's pairwise comparison test. All statistical analyses were carried out with the aid of Statview software (V 5.0; SAS Institute, Cary, NC, USA). Canvas X software was used to build and label the composite illustrations.

## Abbreviations

aa: Amino acid
ac: Anterior commissure
ABC: Avidin-biotin peroxidase complex
Ax: Unmyelinated axon
B: Axonal bouton
BSA: Bovine serum albumin
bv: Blood vessel
Cg1: Anterior cingulate cortex
CLi: Caudal linear raphe nucleus
contra: Contralateral to the injection side
cp: Cerebral peduncle
DA: Dopamine
dcv: Dense-cored vesicle
DAB: 3,3´-diaminobenzidine
Den: Dendrite
DRN: Dorsal raphe nucleus
e: Endothelial cell
FG: Fluorogold
Fm: Forceps minor of the corpus callusum
fr: Fasciculus retroflexus
Hcrt1/OxA: Hypocretin1/OrexinA
IF: Interfascicular subdivision of VTA
IP: Interpeduncular nucleus
ipsi: Ipsilateral to the injection side
ldcv: Large dense-cored vesicle
M2: Secondary frontal cortex
Mc: Muscle cell
mlf: Medial longitudinal fasciculus
MO: Medial orbital cortex
mp: Mammillary peduncle
ob: Olfactory bulb
PAG: Periaqueductal gray
PBP: Parabrachial subdivision of VTA
PL: Prelimbic cortex
PN: Paranigral subdivision of VTA
ri: Rhinal sulcus
RLi: Rostral linear raphe nucleus
RN: Red nucleus
SC: Superior colliculus
SNc: Substantia nigra pars compacta
SNr: Substantia nigra pars reticulata
ssv: Translucent small synaptic vesicles
T: Axon terminal
Ter: Unlabeled axon terminal
TH: Tyrosine hydroxylase
V: Varicosity
un: Unlabeled
VTA: Ventral tegmental area.
